# Exploring the effects of inclusion of dietary fresh *Azolla* on the performance of White Pekin broiler ducks

**DOI:** 10.14202/vetworld.2015.1293-1299

**Published:** 2015-11-14

**Authors:** Parag Acharya, G. P. Mohanty, C. R. Pradhan, S. K. Mishra, N. C. Beura, B. Moharana

**Affiliations:** 1 Department of Livestock Production and Management, National Dairy Research Institute, Karnal, Haryana, India; 2 Department of Livestock Production and Management, College of Veterinary Science and A.H, Orissa University of Agriculture and Technology, Bhubaneswar, Odisha, India; 3 Department of Veterinary, Pharmacology and Toxicology, Madras Veterinary College, Tamil Nadu Veterinary and Animal Sciences University, Chennai, Tamil Nadu, India

**Keywords:** *Azolla*, body weight gain, economic efficiency, feed conversion ratio, White Pekin broilers

## Abstract

**Aim:**

The objective of this study was to investigate the dietary advantages of inclusion of unconventional nutrient source, i.e., *Azolla* in the basal diet of White Pekin broilers.

**Materials and Methods:**

All ducklings were randomly distributed into three treatment groups with three replicates each and each replicate having 12 ducklings and were reared in deep litter system. Groups were subjected to three dietary treatments such as G_1_: Basal diet, G_2_: Basal diet + 5% fresh *Azolla* of basal diet, G_3_: Basal diet + 10% fresh *Azolla* of basal diet. During experimental period, parameters, such as bodyweight, bodyweight gain, feed consumption,feed conversion ratio(FCR), and economic of production,wererecorded.

**Results:**

The result showed that body weights in every week, except the 5^th^ week, did not differ significantly between groups. However, no significant difference was observed between the groups in any week in terms of absolute body weight gain and feed consumption. As regard to FCR, the 10% *Azolla* group (G_3_) showed the best feed efficiency followed by the 5% *Azolla* group (G_2_) and the control group (G_1_). On the basis of profit/bird or profit/kg live weight, each of the *Azolla*-fed group showed a higher economic efficiency than the control.

**Conclusion:**

From the experiment, it was concluded that *Azolla*at 5 or 10% level can be included in the diet to economize the White Pekin broiler farming, and it can be great impetus to poultry farming to include unconventional nutrient source as a dietary supplement.

## Introduction

Among Indian livestock based vocations, poultry farming occupies a special position due to its enormous potential to bring about rapid economic growth with low investment. It is the most profitable enterprise responsible for employment for rural peoples [1]. It has been reported that the modern domestic White Pekin duck performs better than the modern broiler chicken in terms of weight gain and feed efficiency to the same live weight due to genetic improvement [2].

Feeding value of *Azolla* has been experimented by several workers in different poultry species, *viz*. chicken [3,4], ducks [5,6], and quails [7] with promising results in performance. Almost no literature is available on the performance of broiler duck, White Pekin, upon feeding of *Azolla*. The issue looks more pertinent because of the fact that both duck and *Azolla* are water-oriented and that *Azolla* is found ubiquitous in water bodies.

Therefore, the proposed study, i.e. the effect of dietary fresh *Azolla* on the performance of White Pekin broiler ducks has been envisaged to find out the potential effect of fresh *Azolla* on growth performance.

## Materials and Methods

### Ethical approval

Experiments were carried out in accordance with the guidelines laid down by the institute Animal Ethics Committee for the use of animal subjects or that procedures were in compliance with at least the declaration of the National Institute of Health guide for care.

### Location and period of experiment

The experiment was conducted in the department of Livestock Production and Management, College of Veterinary Science and Animal Husbandry, Orissa University of Agriculture and Technology (OUAT), Bhubaneswar. The experimental birds were reared in the Instructional Livestock Farm Complex, OUAT.

### Experimental program

#### Experimental design

About 108-day-old WhitePekin ducklings of either sex were purchased from Central Avian Research Institute (CARI), Bhubaneswar. The ducklings were allowed an adaptationperiod of 2-week along with brooding. All ducklings were weighed and randomly distributed into three treatment groups with three replicates each and each replicate having 12 ducklings, maintaining uniformity in body weight. Ducklings were grown in deep litter system of rearing, and the experimental diets were provided as per BIS [8] specification. The experimental groups were subjected to dietary treatment on completion of 2 weeks of age. The experimental groups were as under.

Group 1: Basal diet (G_1_)
Group 2: Basal diet + 5% fresh *Azolla* of basal diet (G_2_)
Group 3: Basal diet + 10% fresh *Azolla* of basal diet (G_3_)

The diets were made isocaloric and isonitrogenous as per BIS [8] standards.

#### Experimental diets

The ingredient and nutrient compositions of experimental rations are presented in Table-1.

**Table-1:** Ingredient and nutrient composition (% DM) of experimental ration.

Ingredients	G_1_	G_2_	G_3_
Wheat	58	55.5	52.5
Soybean	28	25	23
Rice polish	5	5.5	5.5
Fish meal	6	7	7
*Azolla*	0	5	10
Min. Mix.	2	2	2
Salt	0.3	0.3	0.3
DCP	1	0	0
DL-Meth	0.05	0.05	0.05
Lysine	0.055	0.055	0.055
TM premix	0.1	0.1	0.1
Vitamin B complex	0.015	0.015	0.015
Vitamin ADEK	0.015	0.015	0.015
Ch. chloride	0.05	0.05	0.05
Total	100	100	100
CP	23.3	23.395	23.375
ME	2764	2737.75	2685.25
Ca	1.683	1.449	1.475
Available P	0.626	0.75975	1.01425
Lysine	1.396	1.38225	1.35475
Methionine	0.4346	0.44135	0.43925

DM=Dry matter, CP=Crude protein, ME=Metabolizable energy, DCP=Digestiblecrude protein

The diets for White Pekin broiler ducks were prepared to meet their nutrient requirements as followed in CARI, Bhubaneswar. Experimental feed samples were analyzed for dry matter (DM), crude protein (CP), ether extract, crude fiber, nitrogen free extract (NFE), total ash and acid insoluble ash as per AOAC [9].

#### Cultivation and feeding of Azolla

*Azolla*, required for the preparation of experimental diets was cultivated in the premises of Instructional Livestock Farm Complex, as per the standard procedures [10] with little modification. Pits of size 8ft × 5ft with depths of 10” were dug and spread with polythene sheets to hold the water. In each pit, 10-15 kg of sieved fertile soil was applied uniformly to a thickness of 3”. 2 kg of 2-day-old cow dung was mixed with 10 L of water and poured over it. Single super phosphate was added along with cow dung slurry at a rate of 20-30g/pit. Water was allowed to stand to a depth of 10 cm in the pit. A pure culture of *Azollapinnata* was inoculated at 0.5-1 kg/pit. The pit was completely filled with *Azolla* biomass growth in about 10-15 days. About 10 g of super-phosphate and 500 g of cow dung were added once every 4 days to fertilize the pits and to maintain the production rate of 1 kg/pit every day.

After harvesting, the *Azolla* was rinsed with fresh water for 3-4 times. To remove excess water, it was spread over a net for 3 h. Calculated amount of fresh *Azolla* was offered to birds in separate containers twice daily. At the end of the day, the left over *Azolla* was collected and weighed.

#### Chemical composition of Azolla

The *Azolla* samples were analyzed for the chemical composition such as DM, CP, ether extract, crude fiber, NFE, total ash and acid insoluble ash, as per AOAC. Calcium was determined according to the method modified by Talapatra *et al.*, 1940 [11]. The contents of micro minerals *viz*. zinc, copper, manganese and iron contents in the *Azolla* samples was estimated by collecting 2 g samples and digesting at 120°C using 5 ml conc. HNO_3_ for 1 h using KEL digestion system, India. The digested samples were cooled, and 3 ml of 70% HClO_4_ was added for further digestion at 200°C. The process continued until the contents appeared clear and colorless. The digested samples were filtered into a volumetric flask. The contents of digestion tubes were repeatedly washed with triple distilled water to obtain a complete extract of the mineral. The samples were aspirated into atomic absorption spectrophotometer (ELECO-246) to determine the micro mineral contents.

### Growth performances

#### Body weight and gain in body weight

At the end of each week, the ducklings were weighed individually. Then replicate wise weekly average body weights were calculated. Absolute gains were calculated by subtracting the initial body weight (2^nd^ week) from final body weights of successive weeks.

#### Feed consumption and calculation of feed conversion ratio(FCR)

Daily feed offered to the birds were recorded group wise. The daily group average feed consumption was calculated by subtracting the leftover feed at the next morning from the total feed supplied to the birds on the previous day. Cumulative feed consumption was calculated by adding the feed consumption from the 1^st^ week up to the desired week.


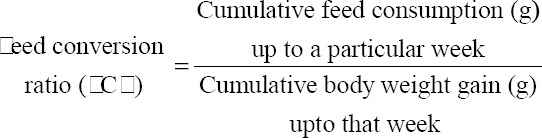


#### Economics of White Pekin broiler duck production

The cost of each diet prepared during broiler duck feeding trial was arrived at by considering the prevailing prices of the constituent feed ingredients, minerals, salts and other additives. Further, by considering the expenditure on ducklings, labor, medicine, etc. to be similar for each treatment, the net profit for each treatment was calculated separately for broiler ducks under different treatments taking into account the sale price of broiler ducks. The relative cost effectiveness of each diet was thus assessed (Table-2).

**Table-2:** Calculated feed cost of different treatment groups.

Ingredients	Cost/kg (Rs)	Cost of ration (Rs)		

		G_1_	G_2_	G_3_
Wheat	20	1160	1110	1050
Soybean	42	1176	1050	966
Rice polish	15	75	82.5	82.5
Fish meal	35	210	245	245
Min. Mix.	60	120	140	140
*Azolla*	01	00	05	10
Salt	15	03	03	03
Total (Rs/100 kg)		2744	2635.5	2496.5
Feed cost/kg		27.44	26.35	24.96

### Statistical analysis

Data obtained from the experiment were subjected to statistical analysis wherever required. The effects of treatments were determined by analyzing the data using one-way ANOVA followed by Duncan’s multiple comparison tests. p<0.05 or <0.01 was considered statistically significant.

## Result

### Chemical composition of *Azolla*

The chemical composition of *Azolla* is presented in Table-3.

**Table-3:** Chemical composition of *Azolla* (DM basis).

Nutrients	Percentage	Nutrients	Percentage
DM	91.07	Calcium	1.10
CP	25.40	Phosphorus	0.55
Crude fiber	14.23	Zinc (ppm)	158.6
Ether extract	2.58	Copper (ppm)	7.33
Total ash	18.76	Manganese (ppm)	83.92
NFE	39.03	Iron (ppm)	283.3

NFE=Nitrogen-free extract, DM=Dry matter, CP=Crude protein

### Body weight and gain in body weight

#### Body weight

The weekly average body weights, for the ducks in the three groups, are presented in Table-4.

**Table-4:** Average weekly body weights (g) of ducks (mean±SE).

Week	Treatment			p value

	G_1_	G_2_	G_3_	
2^nd^ week	249.00±6.81	260.42±6.46	245.5±8.59	0.33
3^rd^ week	545.94±10.77	553.00±10.69	561.64±9.95	0.57
4^th^ week	907.35±16.11	934.54±13.44	954.03±15.44	0.09
5^th^ week**	1199.26^a^±15.29	1240.26^ab^±16.96	1271.67^c^±15.47	0.00
6^th^ week	1483.82±23.59	1533.94±27.11	1560.31±18.17	0.07

** Values bearing different superscripts in a row differ significantly (p≤0.01), SE=Standard error

Body weights in any week, except the 5^th^ week, did not differ significantly (p≥0.05) between groups. In the 5^th^ week, group G_3_ showed significantly (p≤0.01) higher weights than group G_1_ or G_2_, while there was no significant (p≥0.05) difference between the latter two groups.

#### Gain in body weight

The weekly average absolute gains in body weight for three groups of ducks are presented in Table-5. No significant (p≥0.05) difference was observed between the groups in any week.

**Table-5:** Weekly average absolute gains (g) of ducks (mean±SE).

Week	Treatment			p value

	G_1_	G_2_	G_3_	
3^rd^ week	297.24±7.09	292.58±5.56	316.14±5.78	0.08
4^th^ week	658.09±20.37	674.46±15.64	708.53±21.74	0.25
5^th^ week	952.67±17.31	981.21±21.56	1026.17±29.44	0.13
6^th^ week	1233.21±33.11	1273.43±34.76	1314.81±47.95	0.39

SE=Standard error

### Feed consumption and FCR

#### Feed consumption

The weekly cumulative feed consumptions, for the birds in the three groups, are presented in Table-6. No significant (p≥0.05) difference was observed between the groups in any week.

**Table-6:** Cumulative weekly feed consumptions (g) of ducks (mean±SE).

Week	Treatment			p value

	G_1_	G_2_	G_3_	
3^rd^ week	640.00±16.17	635.00±21.79	651.00±13.45	0.81
4^th^ week	1377.00±25.81	1356.00±27.47	1374.00±32.05	0.86
5^th^ week	2218.67±12.00	2179.00±26.65	2190.00±49.96	0.70
6^th^ week	3145.53±25.98	3143.03±76.74	3083.52±82.89	0.77

SE=Standard error

### FCR

The weekly cumulative FCRs, for three groups of ducks, are presented in Table-7.

**Table-7:** FCR of ducks (mean±SE).

Week	Treatment			p value

	G_1_	G_2_	G_3_	
3^rd^ week	2.15±0.05	2.17±0.05	2.06±0.01	0.21
4^th^ week	2.09±0.09	2.01±0.04	1.94±0.02	0.23
5^th^ week**	2.33^c^±0.03	2.22^b^±0.02	2.13^a^±0.01	0.00
6^th^ week**	2.55^b^±0.05	2.47^b^±0.01	2.35^a^±0.03	0.01

Values bearing different superscripts in a row differ significantly (p≤0.05), FCR=Feed conversion ratios, SE=Standard error

There was no significant (p≥0.05) difference between the groups in 3^rd^ and 4^th^ weeks. In the 5^th^ week, each of the *Azolla*-fed group registered a lower value than the control. In the 6^th^ week, the 10% *Azolla* group (G_3_) showed a significantly(p≤0.01) lower value than group G_1_ or G_2_, there being no significant difference (p≥0.05) between the latter two groups. Overall, the 10% *Azolla* group(G_3_) showed the best feed efficiency followed by the 5% *Azolla* group (G_2_) and the control group(G_1_), in that order.

### Economics of production

The economics of production of broiler ducks, for the three groups, are presented in Table-8. The production costs were calculated on the basis of feed cost only.

**Table-8:** Economics of production of ducks.

Parameters	Treatments		

	G_1_	G_2_	G_3_
Total cost of feed consumed/bird (Rs)	86.31	82.73	76.87
Average body weight/bird (in g)	1482	1533	1560
Receipt/bird (Rs)	88.92	91.48	93.6
Profit (Rs) (c-a)	2.61	9.25	16.73
Total cost of feed consumed/kg live weight (Rs)	58.21	54.07	49.28
Difference of cost of feed from control/kg live weight (Rs)	0.00	–4.14	–8.93
Receipt/kg live weight (Rs)	60	60	60
Profit/kg live weight (Rs) (g-e)	1.79	5.93	10.72
Difference in profit/kg live weight over control (Rs)	0	4.14	8.93

Thus, on the basis of profit/bird or profit/kg live weight, each of the *Azolla-*fed group showed higher economic efficiency than the control. Between the *Azolla*-fed groups, group G_3_ showed a higher efficiency than group G_2_.

## Discussion

### Chemical composition of Azolla

The CP content of *Azolla*, estimated in the present study, was (25.40%) which was almost similar to the results obtained by Balaji *et al*. [12]. However, Basak *et al*. [3] reported the higher value of 25.78% CP. The CP contents of *Azolla* estimated by Cherryl *et al*. [13] and Parashuramulu *et al*. [14] were found to be 23.49% and 21.37%, respectively, which were lower than the estimated CP in this study. In the present study, it was found that the crude fiber content obtained was 14.23% which is in line with the value obtained by Parthasarathy *et al*.[15]. The ether extract content of *Azolla* in the present study (2.58%) is almost similar to the earlier observations of Parthasarathy *et al*. [15] and Parashuramulu *et al*. [14], who reported an EE content of 2.3%. The ash content of *Azolla* obtained in this experiment was 18.75%. Parthasarathy *et al*. [15], Basak *et al*.[3], Alalade and Iyayi [16] recorded values almost similar to the present study. TheNFE content of 39.03% recorded in this study is almost similar with the findings of Parthasarathy *et al*. [15] who reported 38.85 to 44.06% NFE in *Azolla*. The levels of calcium and phosphorus in the present study were found to be 1.10% and 0.55%, respectively. The calcium level of *Azolla* obtained in this study was close to the reported value of Alalade and Iyayi [16] but lower than that of the reported value of Cherryl *et al*. [13] who indicated that *Azollamicrophylla* contained 2.58% calcium. Bacerra *et al*. [6] and Balaji *et al*. [12] found 0.4% and 0.44% phosphorus in *Azolla*, which were nearer to the results of the present study. Chemical analysis of dried and ground *Azolla* indicated that it contained 158.6 ppm zinc, 7.33 ppm copper, 83.92 ppm manganese and 283.3 ppm iron on DM basis. However, Alalade and Iyayi [16] reported lower level of zinc and higher level of copper, iron and manganese as observed in the present study. The variations in composition of *Azolla* in comparison to other authors might be due to the soil type, climatic variation, variety of *Azolla*, time of harvest, fertilizer used etc.

### Body weight and gain in body weight

Body weights, for each group, increased progressively in successive weeks till the end of the experiment (6^th^ week). In the 5^th^ week, group G_3_ showed significantly (p≤0.01) higher weights than group G_1_ or G_2_, while there was no significant (p≥0.05) difference between the latter two groups.

This implies that feeding of fresh *Azolla* had beneficial effect on the body weight of ducks without any detrimental effect and a comparable gain in body weight as control group. Provision of free choice diets instead of a complete conventional ration was suggested to have beneficial effects [17]. Choice feeding allows birds a greater opportunity to select the nutrients needed for maintenance and production [18] and will also allow ducks to select correct proportions of the different supplementary feeds to complement their intake. In the course of experiment, it was noticed that ducks had a preference for *Azolla* over the concentrate for which the birds consumed *Azolla* part first. *Azolla*, which is rich in CP and contains high metabolizable energy level, might have improved digestion and also the availability of the dietary nutrients [19]. Furthermore, this might be due to higher bioavailability of essential nutrients present in *Azolla* as it is rich in protein and contains almost all essential amino acids and several growth promoter intermediaries, minerals like calcium, phosphorous, magnesium, potassium, iron, and copper,and the highest nutritive value of this plant derives from the non-structural metabolically active makeup of the plant [20]. Apart from nutrients, *Azolla* also contains certain compounds, such as carotenoids, bio-polymers, and probiotics, which contribute to higher productivity and health of animals [14].

Supplementation of *Azolla* at 10% level exhibited higher growth rate than that in 0 or 5% level. This finding is in agreement with those of Sarria and Preston [21] who reported an increase in growth of broilers when soybean protein was replaced by *Azolla* up to 15% level, when fed at 0%, 10% or 15% level. Seth *et al*. [22] also found higher weight gain in Vanaraja chicken fed *Azolla* at 5% or 10% as compared to those not fed with *Azolla*. Saikia *et al.*, [23] found that highest body weight gain was in 5% inclusion group and lower in 15% inclusion group and the reason mentioned by them as higher level of crude fiber in *Azolla* meal and they conclude that *Azolla* can be included in poultry ration up to 10% level without any significant effect in the performance of broilers for economic effect.

In studies on ducks, the findings in present study corroborate to those of Bacerra *et al*. [6] who reported an increase in daily gains of ducks at 15% replacement of soybean with *Azolla* and that *Azolla* inclusions at 20%, 45%, or 60% level depressed growth rate. Escobin [24] on the other hand, reported that in growing Muscovy ducks, no difference in production efficiency could be noticed by partially replacing traditional rations with *Azolla* at levels of 0%, 20%, 30%, or 40%. These differences might be due to the difference in the duck types used, their basal ration, and environmental conditions.

### Feed consumption and FCR

No significant (p≥0.05) difference was observed between the groups in any week. The lower consumption in *Azolla* fed groups might be due to feeding of fresh *Azolla* with higher moisture content which led to gut fill and hence depressed feed intake. It was also noticed in the course of the experiment that ducks had a preference for fresh *Azolla* to concentrate and consumed the *Azolla* portion first. The beneficial effects of dietary *Azolla* on FCR, as observed in the present study, corroborate those reported by several authors. Basak *et al*. [3] found increased efficiency at 10%, Seth *et al* [22] at 5-15% level, Chichilichi *et al*. [25] at 5% levels of *Azolla* in the diet of chicken. In trials with ducks, Bacerra *et al*. [6] found that by inclusion of *Azolla* from 15% to 60% in diet supplying 15.2-30.3% of the total protein, the FCR decreased with increase in consumption of *Azolla*. Similarly, Lawas *et al*. [26] observed that by feeding Mallard ducks with normal commercial feed allowance of 150g/head/day or 75g/head/day + ad lib. *Azolla*, the FCR was lower for the *Azolla-*fed group. Basak *et al*. [3] reported that by replacing sesame meal with *Azolla* at 5-15% level, the protein and energy efficiency, as well as feed efficiency, decreased with addition of *Azolla* at levels above 5%. Naghshi *et al*. [4] also recorded reduced feed efficiency at *Azolla* inclusion above 5% level. The findings in the present experiment are at variance with those of other authors with respect to the maximal levels of *Azolla* inclusion in diets for improved feed efficiency. Saikia *et al.*, [23] found that *Azolla* did not affect the feed consumption up to 15% inclusion level, and they concluded that *Azolla* has no deleterious effect on palatability of the diets.

On the other hand, several authors have reported that inclusion of *Azolla* at different levels in the diet had no effect on FCR [12,15]. Saikia *et al*. [23] in their study found that 15% *Azolla* fed groups having significantly highest FCR, and they stated the responsible factor as a higher level of crude fiber and tannins.

While Basak *et al*. [3] opined that the higher level of fiber in aquatic plants could be the reason for decreased nutrient utilization and ultimately decreased FCR, Alcantara and Querubin [27], on the other hand, viewed that broilers could readily digest the crude fiber in *Azolla*, so that the digestibility might not have been a limiting factor. They attributed the reason to a decrease in dietary energy density in the ration as reflected from the higher feed consumption in *Azolla*-fed birds. Rai *etal*. [28] concluded that the smaller leaf size was suitable for intake by chicks as well as adults and *Azolla* fed ad lib., when substituted 50% of commercial feed, could provide sufficient nutrient. In the present experiment, the feed consumption for the *Azolla*-fed groups was not increased, nor there any possibilities of decrease in energy density of ration as the rations made isocaloric. Hence, increased feed efficiencies were recorded with increased levels of *Azolla* in the diet.

### Economics of production

Thus, on the basis of profit/bird or profit/kg live weight, each of the *Azolla-*fed group showed higher economic efficiency than the control. Between the *Azolla*-fed groups, group G_3_ (10%) showed a higher efficiency than group G_2_ (5%). Economization of feed cost in poultry by dietary inclusion of *Azolla* at different levels has been reported by several authors, namely, Basak *et al*. [3], Seth *et al*.[22],Chichilichi *et al*.[25],Naghshi *et al*. [4]. In ducks also, the inclusion of *Azolla* in rations has been reported to reduce feed cost significantly [6,24,26,29,30]. Rai *etal*. [28] found in their study that *in situ* cultivation and feeding of *Azolla* to birds under semi-range saved the feed cost by 80%. The higher efficiencies shown by the *Azolla*–fed groups were due to cheap production cost in terms of utilizing unconventional feed source with suffice nutrient content resulting in proper growth and hence entertains the holistic development.

## Conclusion

From the experiment, it was concluded that, inclusion of *Azolla* at 5% or 10% level in the diet improved body weight, gain in body weight and FCR which was at par with standard basal diet. Feed cost of production was substantially minimized by inclusion of *Azolla* at either level. Inclusion of *Azolla* at 10% level showed the maximum economic benefit.

## Authors’ Contributions

The present study was a part of original research work by PA during his MVSc thesis program. GPM conceptualized the aim of the study, designed, planned and supervised the experiment. Collection of samples, execution of experimental study was done by PA, SKM, and NCB. Analysis of data, interpretation of the results and drafting of manuscript was done by PA and CRP. GGP, CRP, SKM, NCB, BM helped in analysis, draft and revision of the manuscript. All authors read and approved the manuscript.
